# Association between weight-adjusted waist index and myopia in adolescents and young adults: results from NHANES 1999–2008

**DOI:** 10.1186/s12886-024-03282-3

**Published:** 2024-01-08

**Authors:** Xu Han Shi, Li Dong, Rui Heng Zhang, Wen Bin Wei

**Affiliations:** 1 Beijing Tongren Eye Center, Beijing Key Laboratory of Intraocular Tumor Diagnosis and Treatment, Beijing Tongren Hospital, Capital Medical University, 1 Dong Jiao Min Lane, Beijing, 100730 China; 2 Beijing Ophthalmology & Visual Sciences Key Lab, Beijing Tongren Hospital, Capital Medical University, Beijing, China; 3 Medical Artificial Intelligence Research and Verification Key Laboratory of the Ministry of Industry and Information Technology, Beijing Tongren Hospital, Capital Medical University, Beijing, China

**Keywords:** Myopia, Obesity, Weight-adjusted waist index, NHANES, Cross-sectional study

## Abstract

**Background:**

Previous studies have indicated a possible link between obesity and myopia, although the results have varied. The objective of this study was to investigate the correlation between a new measure of obesity, the weight-adjusted waist index (WWI), and myopia.

**Method:**

This cross-sectional study included individuals between the ages of 12 and 25 who participated in a noncycloplegic vision examination as part of the National Health and Nutrition Examination Survey (NHANES) conducted from 1999 to 2008. WWI was calculated as waist circumference divided by the square root of body weight. Myopia was characterized by a spherical equivalent (SE) of ≤ − 0.5 diopters (D) and further categorized into mild (-3.00D < SE≤-0.50 D), moderate (-6.00D < SE ≤-3.00 D), or high (SE≤-6.00 D). We utilized a weighted multivariable logistic regression and a generalized additive model to evaluate the correlation between WWI and myopia. Threshold effects were analyzed, and we performed subgroup analysis and interaction tests.

**Results:**

A grand total of 11,180 individuals were registered for the study. Decreased myopia severity was observed with higher WWI, as evidenced by elevated SE (β = 0.098, 95% CI: 0.028–0.167). Individuals in the top tertile of WWI experienced a 19.8% decrease in risk compared to those in the lowest group (OR = 0.802, 95% CI: 0.800-0.804; P for trend < 0.001). Similar associations were observed for high myopia. Gender-specific nonlinear associations were found, with different breakpoints for males (10.774) and females (10.025). In males, a significant positive association was found on the right side of the breakpoint (OR = 1.398, 95% CI: 1.038–1.884), while no significant association was found on the left side. Conversely, among females, a negative association was observed on the left side of the breakpoint (OR = 0.679, 95% CI: 0.512–0.899), whereas no notable correlation was detected on the right side.

**Conclusion:**

Increased WWI level was linked to a lower risk of myopia and high myopia in the overall sample, with gender-specific variations.

## Background

Myopia is the most frequent eye abnormality and mainly develops in childhood and early adulthood. Uncorrected myopia is the prevalent reason for visual impairment [1, 2]. The growing worldwide occurrence of myopia in recent decades has been emphasized as a significant global issue concerning public health. It is estimated that by 2050, approximately 49.8% of the world’s population will likely develop myopia, with 9.8% experiencing high myopia [3]. The harmful effects of myopia go beyond the need to wear corrective lenses. The risk of related complications such as retinal detachment, myopic maculopathy, optic neuropathy, and glaucoma significantly rises with myopia, particularly high myopia, resulting in irreversible vision loss [4, 5]. Several epidemiological studies have shown that high myopia is the leading cause of irreversible blindness in various regions of the world [6–8]. Although both genetic and environmental factors are known to play a role in the progression of myopia, the pathogenesis of myopia is complex and not fully understood [9]. With very limited interventions currently available to effectively prevent myopia [10], it is important to explore the risk factors associated with the development of myopia.

The relationship between weight status or obesity and myopia is not well established. Previous studies on the relationship between obesity and myopia have shown inconsistent results. Some studies have concluded that obesity or overweight is positively associated with myopia [11–14], yet others have found that a lower BMI is associated with the development of myopia [15–17]. Additionally, some studies have found no correlation between weight status and myopia [18–20]. The discrepancies in these results may be due to factors such as different sample sizes in the studies and differences in the definitions of obesity or overweight. Furthermore, traditional factors such as waist circumference, weight, and BMI may also lead to misleading outcomes due to their inability to differentiate between fat mass and muscle [21, 22].

Park et al. proposed the weight-adjusted waist index (WWI) as a novel measure to evaluate obesity. It is defined as the division of waist circumference (WC) by the square root of body weight [23]. It can reflect the composition of fat and muscle mass and serve as a tool to assess central obesity [24]. No research has been conducted on the correlation between WWI and myopia. Exploring the association between WWI and myopia could lead to a greater understanding of the correlation between obesity and myopia.

Based on this view, this study was designed to utilize data from the National Health and Nutrition Examination Survey (NHANES) to thoroughly investigate the correlation between WWI and myopia. The aim was to generate fresh insights for the management and prevention of myopia.

## Methods

### Survey description

The National Centre for Health Statistics (NCHS) conducts NHANES, a survey that evaluates the nutritional and overall health of individuals of all ages in the United States. To obtain a sample that accurately represents the noninstitutionalized civilian population in the United States, a sophisticated multistage probability design was utilized [25]. Participants provided demographic, socioeconomic, and medical information during an in-home interview. A mobile examination center (MEC) was used to conduct physical and laboratory examinations. The detailed design and data of the NHANES study are available to the public at the following website: https://www.cdc.gov/nchs/nhanes/index.htm.

The NHANES study protocols were approved by the Research Ethics Review Board of the NCHS, in accordance with the ethical guidelines of the 1975 Declaration of Helsinki. All survey participants and/or their guardians provided their written informed consent. The present investigation adhered to the guidelines of Strengthening the Reporting of Observational Studies in Epidemiology (STROBE) for the reporting of cross-sectional studies [26].

### Study population

**Fig. 1:**
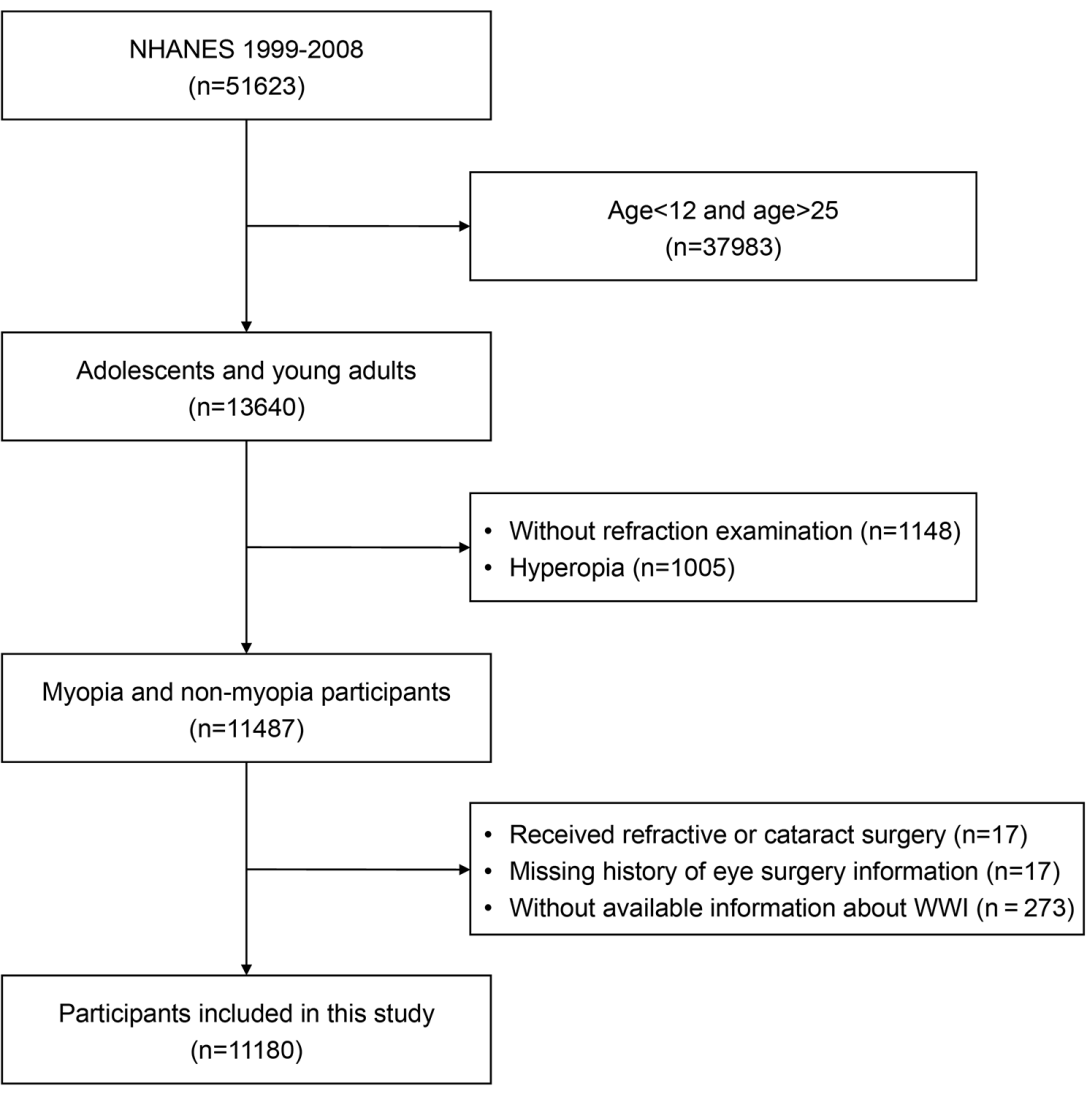
Flow chart of the sample selection process

For this research, the data spanning from 1999 to 2008 were used because the ophthalmic evaluation was available only in the period of 1999 to 2008. Because refraction measurement data were only available for participants aged 12 or older, the current analyses were restricted to individuals aged 12 to 25 years, with the upper age limit accounting for late-onset myopia [27]. Our analysis included individuals who underwent refraction measurements and had complete data on WWI. In our final analysis, we included 11,180 eligible participants after excluding the participants younger than 12 and older than 25 years (*n* = 37,983), missing refraction measurement results (*n* = 1148), hyperopia (defined as refractive error > 0.5 D in any eye, *n* = 1005), received cataract or refractive surgery (*n* = 17), missing history of eye surgery information (*n* = 17), and individuals without available information about WWI (*n* = 273) (Fig. 1).

### Assessment of myopia

All participants underwent a noncycloplegic vision examination. The refractive status of the eye was objectively evaluated using an autorefractor (Nidek ARK-760 A, Nidek Co. Ltd., Gamagori, Japan). Each eye was measured three times consecutively, and the median values of the sphere, cylinder, and axis were recorded. The spherical equivalent (SE) was calculated as the sphere + 1/2 cylinder. Due to the high correlation between the SE values of the right and left eyes, we used only the right eye for the analysis in this study. Myopia was characterized as a SE≤-0.50 diopters (D). Specifically, myopia was categorized into mild (-3.00 D < SE≤-0.50 D), moderate (-6.00D < SE ≤-3.00 D), or high (SE≤-6.00 D) myopia.

### Evaluation of WWI

WWI is a novel anthropometric indicator that utilizes waist circumference and weight to assess the level of central obesity. In general, an elevated WWI corresponds to a higher degree of central adiposity. Anthropometry measurements were conducted in MECs by qualified health technicians. To determine the WWI for each participant, the WC (cm) was divided by the square root of their weight (kg). During the analysis, we considered WWI as a continuous factor and categorized individuals into three groups for further examination, according to the WWI tertiles. Our study utilized WWI as a variable for exposure.

### Covariates of interest

Potential covariates between WWI and myopia included age, gender, ethnicity, education level (≤ 9th grade, 9–12th grade, high school grade, and college or above), body mass index (BMI, kg/m^2^), family poverty income ratio (PIR), and TV and computer use time (hours per day), which were adjusted for in the present study. Through the analysis of vitamin D levels, we attempted to adjust for time spent outdoors. Ethnicity, education level, PIR, and TV and computer use time were ascertained by questionnaire. Weight and height measurements were taken in the MEC by trained health professionals, and BMI was calculated. Serum vitamin D levels were determined by measuring serum concentrations of 25(OH)D, which was only performed in NHANES 2001–2006. Consequently, serum vitamin D concentration was analyzed separately.

### Statistical analysis

To account for the complex sampling design of the NHANES, statistical analyses were performed following CDC guidelines and utilizing suitable weights for the NHANES sampling [25].

According to the recommendations from the NHANES official website, we incorporate weights in our analyses. The method for calculating these weights follows the guidelines provided on the following website: https://wwwn.cdc.gov/nchs/nhanes/tutorials/Weighting.aspx. Weighted Student’s t tests were used to assess the differences among categories of myopia levels (mild, moderate, and high) in descriptive analyses, for continuous variables. Additionally, weighted chi-square tests were employed for categorical variables. In order to represent the extensive, nationally representative sample, inferential statistics were employed due to the intricate and multi-level probability sampling design of NHANES. Consequently, linear regression analyses are used to summarize continuous variables as means with standard errors, while logistic regression analyses are employed to display categorical parameters as proportions.

Two separate multivariable regression models were utilized to examine the correlation between WWI and myopia, taking into consideration the sampling weights. Sex, age, and ethnicity were taken into account when adjusting Model (1) Sex, age, ethnicity, education level, BMI, PIR, TV and computer usage duration, and serum vitamin D level were taken into account when adjusting Model (2) After converting WWI into a categorical variable (divided into three equal groups), we conducted additional sensitivity analysis to evaluate its reliability.

In both the overall sample and each subgroup, a generalized additive model (GAM) and fitting smooth curves were employed to establish the nonlinear correlation between WWI and myopia. Covariates included sex, age, ethnicity, education level, BMI, PIR, TV and computer usage duration, and serum vitamin D level. The evaluation was conducted using smoothing splines. When a nonlinear correlation was identified, a segmented regression model was utilized to fit each interval and threshold effects are determined. A log-likelihood ratio test was conducted to ascertain the presence of a threshold by comparing the nonsegmented one-line model with the segmented regression model. The breakpoint (K) connecting the segments was determined through a two-step recursive approach, utilizing the model that offers the highest probability. In addition, the association between WWI and myopia and high myopia was examined through stratified multivariable logistic regression models, considering factors such as sex and age. Furthermore, these categorized variables were regarded as possible modifiers of effects, and an interaction term was included through the likelihood ratio test to assess the diversity of relationships among different subgroups.

Use the median to fill in missing values for continuous variables and the plural to fill in missing values for categorical variables. R version 4.1.3 (The R Foundation) and Empower software (X&Y solutions, Inc., Boston, MA, United States) were utilized for all the analyses. A significance level of less than 0.05 was used to determine statistical significance in both directions.

## Result

### Participants’ initial characteristics

In this cross-sectional study, a grand total of 11,180 individuals were enrolled, with males accounting for 50.82% and females accounting for 49.18% (based on weighted proportions). The weighted proportions of emmetropes, mild myopia, moderate myopia, and high myopia patients were 51.84%, 34.16%, 10.74%, and 3.27%, respectively. Participants with larger refractive errors tended to be of advanced age, more inclined towards the female gender, belonging to non-Hispanic White or other racial backgrounds, and had higher levels of education compared to those with emmetropes. (Table 1). In the emmetropes group, mild myopia group, moderate myopia group, and high myopia group, the mean (standard error) WWI values were 10.24 (0.03), 10.22 (0.02), 10.21 (0.02), and 10.13 (0.05), respectively. Participants with high myopia had a smaller WWI than those with emmetropes. However, there was no statistically noteworthy disparity in WWI among the various myopia categories (*p* = 0.084) (Table 1).

**Table 1:** Cross-sectional characteristics of participants, NHANES 1999–2008

	No. of available cases	Overall (*n* = 11,180)	Emmetropes (*n* = 5909)	Mild myopia (*n* = 3961)	Moderate myopia (*n* = 1034)	High myopia (*n* = 276)	*P* value
Weighted proportion (%)	11,180		51.84 (0.87)	34.16 (0.74)	10.74 (0.58)	3.27 (0.31)	
SE (D)	11,180	-1.13 (0.04)	0.02 (0.01)	-1.30 (0.02)	-4.14 (0.04)	-7.84 (0.16)	< 0.001
Age (year)	11,180	18.32 (0.07)	18.12 (0.09)	18.32 (0.11)	18.83 (0.19)	19.97 (0.28)	< 0.001
Sex (%)	11,180						< 0.001
Male	5542	50.82 (0.58)	53.33 (0.87)	51.06 (1.15)	40.80 (2.12)	41.45 (3.94)	
Female	5638	49.18 (0.58)	46.67 (0.87)	48.94 (1.15)	59.20 (2.12)	58.55 (3.94)	
Ethnicity (%)	11,180						< 0.001
Mexican American	3636	12.01 (0.94)	11.99 (0.90)	13.61 (1.22)	8.35 (1.21)	7.57 (1.51)	
Other Hispanic	607	6.63 (0.82)	6.90 (1.20)	6.61 (0.88)	5.95 (1.10)	4.86 (1.85)	
Non-Hispanic White	3230	61.27 (1.53)	61.73 (1.64)	58.98 (1.76)	65.09 (2.50)	65.37 (3.73)	
Non-Hispanic Black	3221	13.81 (1.02)	14.11 (1.08)	14.51 (1.19)	11.50 (1.37)	9.51 (1.70)	
Other Race	486	6.27 (0.54)	5.27 (0.66)	6.28 (0.67)	9.11 (1.53)	12.70 (3.27)	
Education level (%)	11,176						< 0.001
< 9th Grade	4187	27.34 (0.69)	29.28 (0.98)	27.98 (1.07)	21.50 (1.75)	9.21 (1.79)	
9–12th Grade	4101	30.04 (0.76)	31.43 (1.07)	28.79 (1.00)	28.61 (1.98)	25.78 (3.56)	
High School Grade	1358	16.08 (0.70)	15.95 (0.93)	16.53 (1.01)	14.81 (1.81)	17.54 (2.79)	
College or above	1530	26.54 (0.99)	23.35 (1.25)	26.70 (1.31)	35.07 (2.34)	47.46 (3.60)	
BMI (kg/m2)	11,180	24.65 (0.11)	24.50 (0.14)	24.83 (0.15)	24.89 (0.28)	24.60 (0.41)	0.042
WC (cm)	11,180	84.81 (0.30)	85.41 (0.78)	85.19 (0.41)	84.47 (0.38)	84.15 (1.05)	0.068
WWI (cm/√kg)	11,180	10.22 (0.02)	10.24 (0.03)	10.22 (0.02)	10.21 (0.02)	10.13 (0.05)	0.084
PIR	10,329	2.42 (0.04)	2.38 (0.05)	2.41 (0.05)	2.68 (0.09)	2.42 (0.18)	< 0.001
TV and computer use (hours per day)	7074	4.13 (0.04)	3.87 (0.15)	4.07 (0.06)	4.08 (0.09)	4.19 (0.05)	< 0.001
Vitamin D level, nmol/L^a^	7910	62.95 (0.78)	63.43 (0.83)	61.98 (0.94)	63.90 (1.35)	62.29 (2.78)	0.036

Data were weighted estimates and expressed as the mean (standard error) or percentage (%)

^a^The serum vitamin D level was measured only in NHANES 2001–2006

SE: Spherical equivalent, BMI: body mass index, WC: waist circumference, WWI: weight-adjusted-waist index, PIR: poverty income ratio

### The correlation between WWI and myopia

The correlation between WWI and myopia is shown in Table 2. In both the minimally adjusted and fully adjusted models, we discovered a correlation between a higher WWI and an elevated SE. After adjusting for all variables, a single increment in WWI score corresponded to a 0.098 D increase in SE (β = 0.098, 95% CI: 0.028–0.167). Even after WWI was classified as a tertile, this association remained statistically significant. In the fully adjusted model, participants in Tertile 3 of WWI had a notable 0.234 D increase in SE compared to Tertile 1, which had the lowest WWI. (Tertile 3: β = 0.234, 95% CI: 0.109–0.359).

**Table 2:** Association between weight-adjusted waist index and myopia

Weight-adjusted-waist index group	Minimally adjusted model (Model 1) ^a^	Fully adjusted model (Model 2) ^b^
**SE** ^**c**^ **(D)/β** ^**d**^ **(95% CI** ^**e**^ **)**		
Continuous	0.067 (0.018, 0.115)	0.098 (0.028, 0.167)
**Categories**		
Tertile 1	Reference	Reference
Tertile 2	0.048 (-0.039, 0.134)	0.101 (-0.001, 0.203)
Tertile 3	0.148 (0.054, 0.241)	0.234 (0.109, 0.359)
P for trend	0.002	< 0.001
**Myopia/OR** ^**f**^ **(95% CI)**		
Continuous	0.967 (0.966, 0.967)	0.922 (0.921, 0.924)
**Categories**		
Tertile 1	Reference	Reference
Tertile 2	0.968 (0.967, 0.970)	0.923 (0.922, 0.925)
Tertile 3	0.910 (0.908, 0.911)	0.802 (0.800, 0.804)
P for trend	< 0.001	< 0.001
**Mild myopia /OR (95% CI)**		
Continuous	0.988 (0.987, 0.989)	0.930 (0.929, 0.931)
**Categories**		
Tertile 1	Reference	Reference
Tertile 2	0.974 (0.972, 0.975)	0.924 (0.923, 0.926)
Tertile 3	0.933 (0.931, 0.935)	0.804 (0.802, 0.806)
P for trend	< 0.001	< 0.001
**Moderate myopia/OR (95% CI)**		
Continuous	0.945 (0.944, 0.946)	0.956 (0.954, 0.958)
**Categories**		
Tertile 1	Reference	Reference
Tertile 2	0.975 (0.973, 0.978)	0.965 (0.962, 0.967)
Tertile 3	0.920 (0.917, 0.922)	0.899 (0.896, 0.902)
P for trend	< 0.001	< 0.001
**High myopia/OR (95% CI)**		
Continuous	0.713 (0.711, 0.715)	0.646 (0.644, 0.649)
**Categories**		
Tertile 1	Reference	Reference
Tertile 2	0.836 (0.833, 0.829)	0.757 (0.754, 0.761)
Tertile 3	0.556 (0.553, 0.558)	0.442 (0.439, 0.445)
P for trend	< 0.001	< 0.001

Data were weighted estimates

In sensitivity analysis, the weight-adjusted waist index was converted from a continuous variable to a categorical variable (tertiles)

^a^Model 1: adjusted for sex, age and race

^b^Model 2: adjusted for sex, age, race, education level, body mass index, serum vitamin D level, poverty income ratio, and TV and computer use time

^c^SE: Spherical equivalent

^d^β: effect size

^e^95% CI: 95% confidence interval

^f^OR: odds ratio

Our findings indicated that a higher WWI was associated with a lower probability of myopia. After making extensive adjustment, individuals with an additional WWI unit experienced a 7.8% lower risk of developing myopia (Model 2: OR = 0.922, 95% CI: 0.921–0.924). After WWI was stratified into tertiles, this association remained statistically significant. Compared with the individuals in the lowest WWI tertile, those in the highest WWI tertile experienced a noteworthy reduction of 19.8% in the risk of myopia (OR = 0.802, 95% CI: 0.800-0.804; P for trend < 0.001) (Table 2).

After grouping myopia by severity, we conducted further analysis and found a statistically significant negative association between WWI and the likelihood of having mild myopia, moderate myopia, and high myopia, and this association persisted across both of our models. During the comprehensive adjustment analysis, individuals with a WWI that was one unit higher experienced a 7.0% decrease in the likelihood of developing mild myopia (OR = 0.930, 95% CI: 0.929–0.931), a 4.4% decrease in the likelihood of developing moderate myopia (OR = 0.956, 95% CI: 0.954–0.958), and a 35.4% decrease in the likelihood of developing high myopia (OR = 0.646, 95% CI: 0.646–0.649). As WWI was categorized into tertiles, people in the top group demonstrated a 19.6% reduction in high myopia risk (OR = 0.804, 95% CI 0.802–0.806; P for trend < 0.001), a 10.1% reduction in high myopia risk (OR = 0.899, 95% CI 0.896–0.902; P for trend < 0.001), and a significant 55.8% reduction in high myopia risk (OR = 0.442, 95% CI 0.439–0.445; P for trend < 0.001) compared to those in the lowest group. (Table 2).

**Fig. 2:**
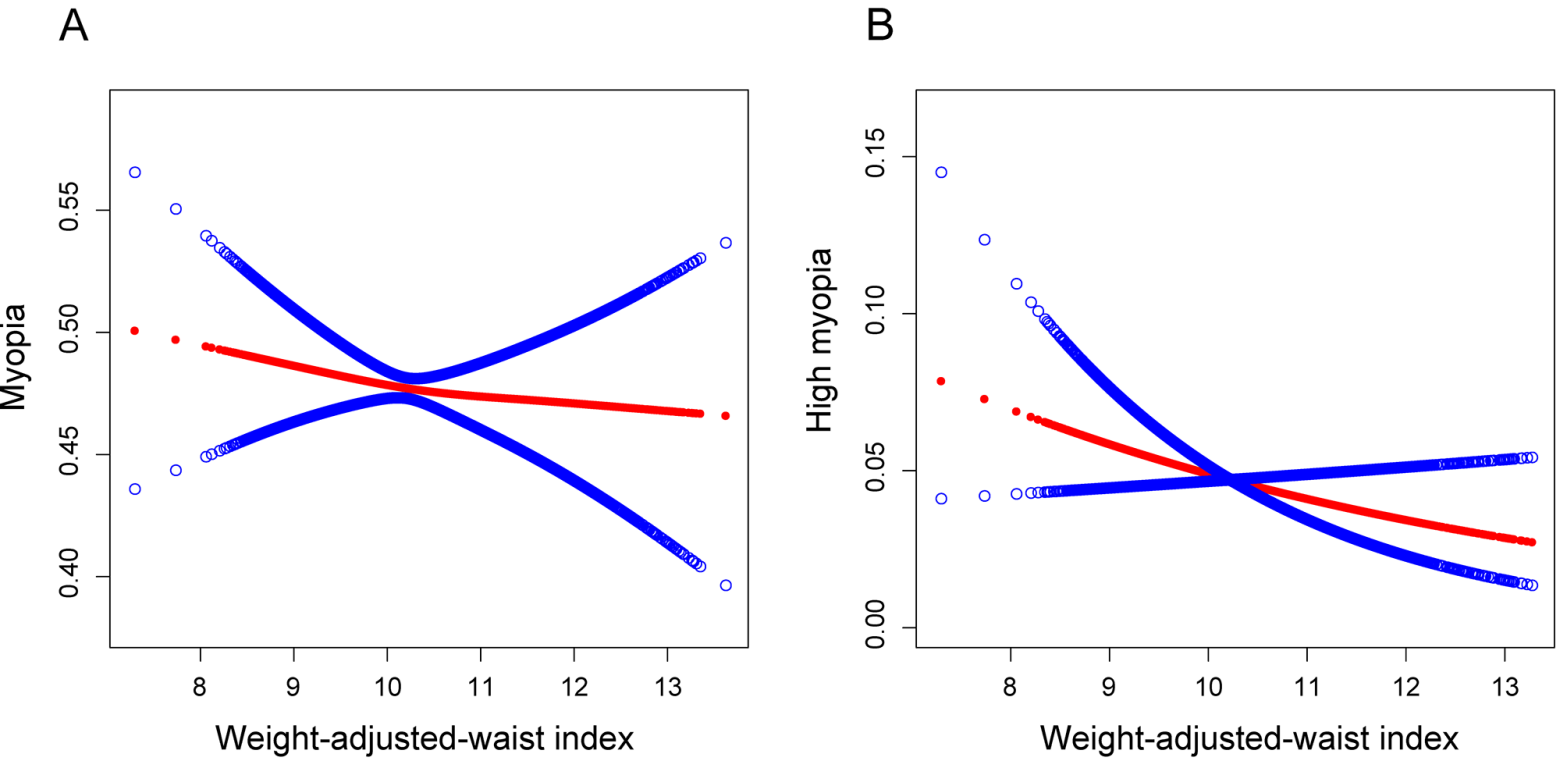
A linear relationship between WWI and myopia or high myopia was detected by the generalized additive model. **A**: Smooth curve fitting for weight-adjusted-waist index (WWI) and myopia. **B**: Smooth curve fitting for WWI and high myopia

Additionally, the smooth curve fitting results also demonstrated that there was a negative correlation between WWI and the occurrence of myopia (Fig. 2A) or high myopia (Fig. 2B) in the total sample of individuals.

### Subgroup analysis

To ascertain if the correlation between WWI and myopia remained consistent throughout the entire population and to identify potential variations in different population groups, we performed a subgroup analysis and interaction test, stratifying by age and sex. Our findings demonstrated that there was no dependence. According to Table 3, there was no significant interaction across sex and age (all P for interaction > 0.05). In all age groups and among females, the linear negative association remained significant (Table 3). Significant interactions were observed between sex (P for interaction = 0.009) and age (P for interaction < 0.001) in the correlation between WWI and high myopia. In all age groups and among women, we observed a negative correlation. In summary, our study findings indicate that the link between WWI and myopia remains unaffected by gender and age. In contrast, the relationship between WWI and high myopia appeared to depend on both age and sex. It is possibly appropriate for women and all age groups (Table 3).

**Table 3:** Subgroup analysis for the association between WWI and myopia or high myopia

Subgroup	OR (95% CI)	P for interaction
**For myopia**		
**Sex**		0.108
Male	1.026 (0.916, 1.149)	
Female	**0.907 (0.824, 0.988)**	
**Age**		0.080
< 20 years	**0.970 (0.969, 0.972)**	
≥ 20 years	**0.868 (0.866, 0.869)**	
**For high myopia**		
**Sex**		**0.009**
Male	1.220 (0.821, 1.812)	
Female	**0.636 (0.466, 0.867)**	
**Age**		**< 0.001**
< 20 years	**0.940 (0.936, 0.945)**	
≥ 20 years	**0.501 (0.498, 0.503)**	

Sex, age, race, education level, body mass index, serum vitamin D level, poverty income ratio, and TV and computer use time were adjusted

In the subgroup analyses, the model was not adjusted for the stratification variable itself

OR: odds ratio; 95% CI: 95% confidence interval

### Nonlinear correlation between WWI and myopia in various sexes groups

**Fig. 3:**
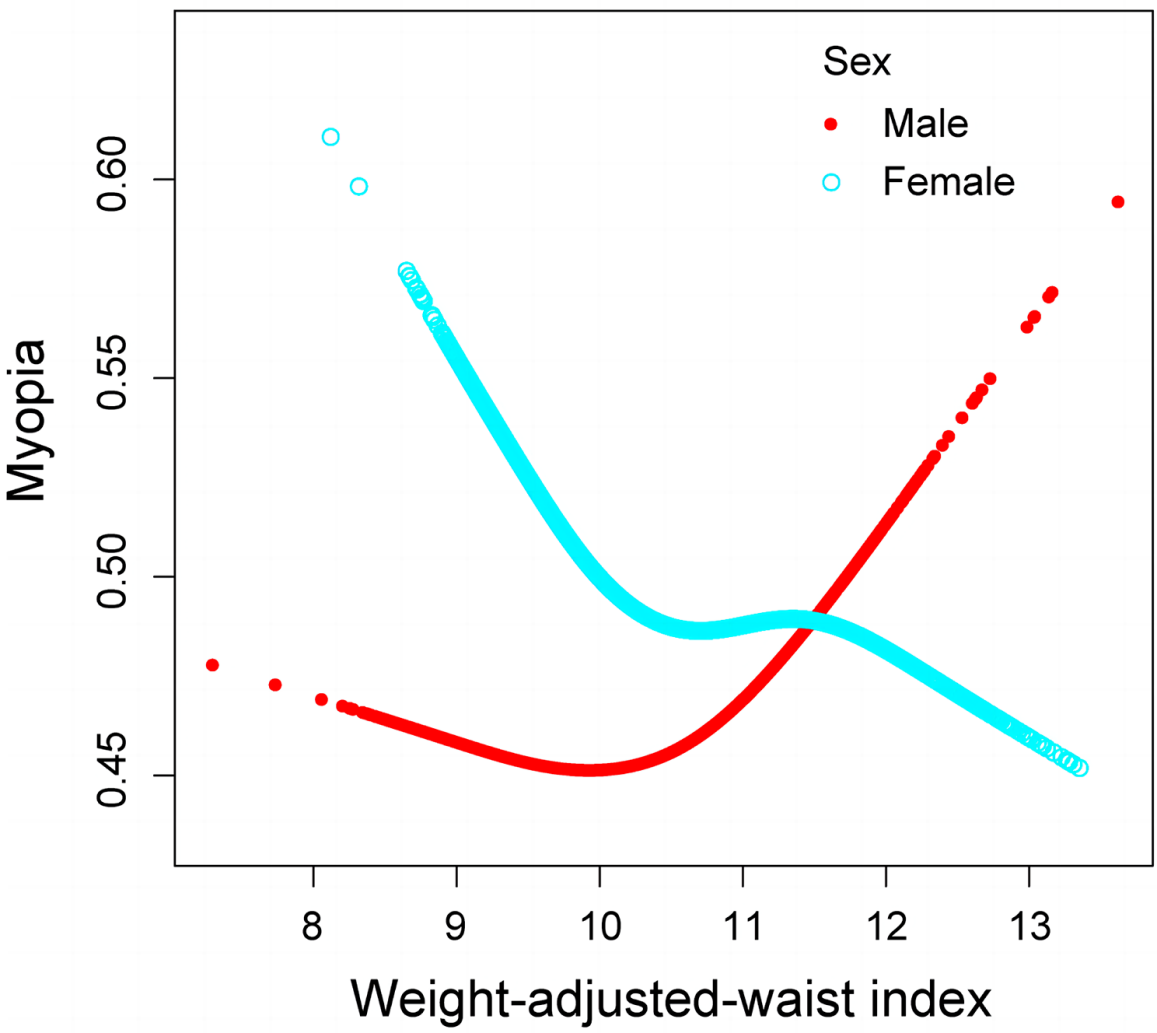
Nonlinear relationship between WWI and myopia in different sexes by the generalized additive model

In addition, the nonlinearity for each stratification was resolved using GAM and smooth curve fittings. When age was taken into account, there was no nonlinear correlation found between WWI and myopia. However, it was observed that both male and female participants exhibited a nonlinear relationship in the resultant curve of gender (Fig. 3). The males’ breakpoint (K) was determined to be 10.774, while for females it was calculated as 10.025, based on the two-piecewise linear regression model. Among males, a positive correlation between WWI and myopia on the right of the breakpoint was observed (OR = 1.398, 95% CI: 1.038–1.884). Nevertheless, there was no statistically significant relationship on the left (OR = 0.938, 95% CI: 0.818–1.077). For female individuals, a negative relationship between WWI and myopia was found to the left of the breakpoint (OR = 0.679, 95% CI: 0.512–0.899), whereas no statistically significant correlation was found on the right side (OR = 0.987, 95% CI: 0.875–1.112) (Table 4).

**Table 4:** Threshold effect analysis of WWI on myopia using a two-piecewise linear regression model

	Male	Female
**Fitting by standard linear model**		
OR (95% CI)	1.026 (0.916, 1.149)	**0.907 (0.824, 0.988)**
*P* value	0.656	**0.046**
**Fitting by two-piecewise linear model**		
Breakpoint (K)	10.774	10.025
WWI < K	0.938 (0.818, 1.077) 0.366	**0.679 (0.512, 0.899)** **0.007**
WWI > K	**1.398 (1.038, 1.884)** **0.028**	0.987 (0.876, 1.112) 0.829
Logarithmic likelihood ratio test *P* value	**0.022**	**0.027**

Sex, age, race, education level, body mass index, serum vitamin D level, poverty income ratio, and TV and computer use time were adjusted

The model is not adjusted for the stratification variable itself

OR: odds ratio; 95% CI: 95% confidence interval; WWI: weight-adjusted-waist index

## Discussion

The aim of this research was to evaluate the correlation between WWI and myopia in American adolescents and young adults. In our study that included 11,180 participants, we noticed a decreased likelihood of myopia and high myopia among individuals with higher WWI. Subgroup analyses and interaction tests revealed variations in this association between different gender populations. Males showed a nonlinear correlation between WWI and myopia. On the right side of the breakpoint, there was a positive relationship, while no significant association was found on the left side. In contrast, females displayed a different pattern. On the left side, there was a detrimental association, while the correlation on the right side lacked significance.

To our understanding, this study is the initial attempt to assess the connection between WWI and myopia; it highlights an inverse correlation between WWI levels and the risk of myopia and high myopia as well as the differences in this correlation among different gender populations. Previous studies have predominantly focused on body mass index (BMI) as a measure of obesity, and the correlation between obesity and myopia has yielded inconsistent results. Some studies reported a decreased prevalence of myopia associated with higher BMI [15, 16], while others have yielded contradictory findings [12–14]. And some studies have shown no association between obesity and myopia [18–20]. Interestingly, a recent study [28] revealed that both low weight and overweight were linked to higher risk of myopia and high myopia, with the lowest risk in healthy-weight adolescents. The differences in these research findings may be attributed to various factors, with a crucial factor being the inability of traditional evaluation indicators such as BMI, weight, and WC to distinguish between muscle mass and adipose tissue.

The WWI is a recently introduced obesity index that is already widely used in number of areas [29–31], and was identified as a more inclusive measure of obesity, specifically highlighting central obesity [23]. Kim et al. [32] found that various indicators of fat mass showed a positive correlation with WWI, whereas indicators of muscle mass exhibited a negative correlation with WWI. These findings have been corroborated by studies involving diverse ethnic groups, affirming the credibility and potential utility of WWI [24]. Notably, compared to some complex mathematical formulas, WWI employs a relatively simple calculation formula, which facilitates its use in routine examinations. Therefore, WWI was employed to evaluate the degree of authentic obesity in this investigation.

We observed that a higher WWI was linked to a reduced risk of myopia and high myopia (Fig. 2). Sensitivity analysis using WWI tertiles confirmed this trend. However, subgroup analysis and smooth curve fittings revealed notable gender-specific associations between WWI and myopia. We found that for males, when WWI exceeded 10.774, the risk of myopia significantly increased with increasing WWI. In contrast, for females, when WWI was below 10.025, the risk of myopia decreased with increasing WWI, stabilizing after surpassing this threshold (Fig. 3). Therefore, considering a comprehensive perspective of controlling overall health risks and reducing the risk of myopia, maintaining a moderate WWI is of utmost importance for both men and women.

The underlying mechanisms of the correlation between WWI and myopia are not yet fully understood. Regarding the relationship between weight status and myopia, there have been conflicting research results and various plausible theoretical hypotheses. First, there may be differences in environmental factors between overweight and normal-weight individuals, including the time spent using electronic devices such as televisions or computers, outdoor activity time, educational stress, family income level, and other factors that could potentially influence myopia [9, 10]. Additionally, Gunes et al. [32] reported that, unlike the accumulation of other adipose tissues in the body, retrobulbar fat is constrained by the orbital space. In obese individuals, an increase in retrobulbar fat can slow axial growth and eye expansion. Therefore, obese individuals often tend to have a higher prevalence of hyperopia and a shorter vitreous chamber. On the other hand, some researchers suggest that insulin resistance in obesity may be associated with the development of myopia. Insulin resistance leads to elevated insulin and insulin-like growth factor 1 (IGF-1), which is an effective growth stimulator and may induce scleral tissue growth and elongation of the eye axis, thus leading to the development of myopia [33, 34].

We propose that the relationship between WWI and myopia may be the result of the combined interaction of the aforementioned factors. The association between WWI and myopia observed in males and females differs, indicating a potential gender difference in the emmetropization mechanism. One possible explanation is the variation in fat distribution between males and females. Males tend to accumulate fat more readily in the abdominal region, whereas females exhibit less abdominal fat accumulation [35, 36]. Therefore, in females, an increase in WWI is more likely to lead to greater orbital fat deposition, resulting in a stronger inhibition of axial elongation and myopia development. In contrast, in males, with a preferential accumulation of fat in the abdominal region, an increase in WWI may not exhibit important changes in orbital fat deposition, leading to less pronounced inhibition of axial elongation. Additionally, in conjunction with the effect of insulin resistance, it is possible that this combination ultimately leads to the progression of myopia in males.

Our study has several strengths. This is the initial study that aims to evaluate the correlation between the novel obesity index WWI and myopia and high myopia, providing a more in-depth exploration of the association between obesity and myopia as well as analyzing the underlying factors. Moreover, our study is based on a large nationally representative sample with consideration of sample weights, making the findings widely applicable to the general population of the United States. Additionally, we further investigated the nonlinear relationship among participants of different sexes and identified reliable thresholds, offering new insights for the prevention and control of myopia.

However, this study also has some limitations. Because the data of our research was obtained from a public dataset with a cross-sectional design, we cannot establish a causal relationship between WWI and myopia. Furthermore, despite our efforts to account for certain variables, there may still be other factors that could impact the findings, including but not limited to parental history of myopia, age of onset of myopia, and outdoor activity time. Since NHANES lacks these data, we were unable to include these covariates, potentially compromising the completeness and accuracy of the results. We attempted to correct for outdoor activity time by analyzing vitamin D levels, but vitamin D levels only serve as an indicator of recent outdoor activity and may not fully reflect long-term outdoor activity [37, 38]. Third, the study of axial length is also meaningful for myopia research; however, NHANES does not include data on axial length, thus preventing us from analyzing the relationship between WWI and axial length. Additionally, the refractive data in NHANES is not obtained through cycloplegic refraction, which may result in an inaccurate assessment of myopia. We hope to further explore with data obtained through cycloplegic refraction in future studies for a more accurate evaluation.

In conclusion, our study indicated that in the overall sample, an increase in WWI was associated with a reduced risk of myopia and high myopia. However, the relationship between WWI and myopia showed clear gender specificity. In males, there was a nonlinear association of a positive nature between WWI and myopia, while in females, a nonlinear association of a negative nature was observed between WWI and myopia. In both males and females, WWI exhibited an important threshold effect on myopia. Considering the comprehensive perspective of controlling overall health risks and reducing the risk of myopia, maintaining a moderate WWI is crucial for both males and females.

## Data Availability

A public dataset was analyzed in this study. This data can be found here: https://www.cdc.gov/nchs/nhanes/.

## Abbreviations

WWI: Weight-adjusted waist index
WC: Waist circumference
NHANES: The National Health and Nutrition Examination Survey
NCHS: The National Centre for Health Statistics
MEC: Mobile examination center
SE: Spherical equivalent
BMI: Body mass index
PIR: Family poverty income ratio
GAM: Generalized additive model
IGF-1: Insulin-like growth factor 1

## References

[1] Baird PN, Saw S-M, Lanca C, Guggenheim JA, Smith Iii EL, Zhou X, Matsui K-O, Wu P-C, Sankaridurg P, Chia A et al. Myopia. *Nat Rev Dis Primers* 2020, 6(1):99. 10.1038/s41572-020-00231-4 33328468

[2] Chua J, Wong TY (2016). Myopia-the Silent Epidemic that should not be ignored. JAMA Ophthalmol.

[3] Holden BA, Fricke TR, Wilson DA, Jong M, Naidoo KS, Sankaridurg P, Wong TY, Naduvilath TJ, Resnikoff S (2016). Global prevalence of myopia and high myopia and temporal trends from 2000 through 2050. Ophthalmology.

[4] Tideman JWL, Snabel MCC, Tedja MS, van Rijn GA, Wong KT, Kuijpers RWAM, Vingerling JR, Hofman A, Buitendijk GHS, Keunen JEE (2016). Association of axial length with risk of uncorrectable visual impairment for europeans with myopia. JAMA Ophthalmol.

[5] Wong TY, Ferreira A, Hughes R, Carter G, Mitchell P. Epidemiology and disease burden of pathologic myopia and myopic choroidal neovascularization: an evidence-based systematic review. Am J Ophthalmol 2014, 157(1). 10.1016/j.ajo.2013.08.010 24099276

[6] Xu L, Wang Y, Li Y, Wang Y, Cui T, Li J, Jonas JB. Causes of blindness and visual impairment in urban and rural areas in Beijing: the Beijing Eye Study. *Ophthalmology* 2006, 113(7):1134.e1131-1134.1111. 10.1016/j.ophtha.2006.01.035 16647133

[7] Liang YB, Friedman DS, Wong TY, Zhan SY, Sun LP, Wang JJ, Duan XR, Yang XH, Wang FH, Zhou Q (2008). Prevalence and causes of low vision and blindness in a rural Chinese adult population: the Handan Eye Study. Ophthalmology.

[8] Bikbov MM, Kazakbaeva GM, Zainullin RM, Gilmanshin TR, Nuriev IF, Zaynetdinov AF, Yakupova DF, Uzianbaeva YV, Panda-Jonas S, Mukhamadieva SR (2020). Prevalence and causes of vision impairment and blindness in the Russian ural eye and medical study. Sci Rep.

[9] Morgan IG, Ohno-Matsui K, Saw S-M. Myopia. *Lancet* 2012, 379(9827):1739–1748. 10.1016/S0140-6736(12)60272-4 22559900

[10] Morgan IG, French AN, Ashby RS, Guo X, Ding X, He M, Rose KA (2018). The epidemics of myopia: Aetiology and prevention. Prog Retin Eye Res.

[11] Lee S, Lee H-J, Lee KG, Kim J (2022). Obesity and high myopia in children and adolescents: Korea National Health and Nutrition Examination Survey. PLoS ONE.

[12] Harrington SC, Stack J, O’Dwyer V (2019). Risk factors associated with myopia in schoolchildren in Ireland. Br J Ophthalmol.

[13] Tideman JWL, Polling JR, Hofman A, Jaddoe VW, Mackenbach JP, Klaver CC (2018). Environmental factors explain socioeconomic prevalence differences in myopia in 6-year-old children. Br J Ophthalmol.

[14] Kim H, Seo JS, Yoo W-S, Kim G-N, Kim RB, Chae JE, Chung I, Seo S-W, Kim SJ (2020). Factors associated with myopia in Korean children: Korea National Health and nutrition examination survey 2016–2017 (KNHANES VII). BMC Ophthalmol.

[15] Saw S-M, Chua W-H, Hong C-Y, Wu H-M, Chia K-S, Stone RA, Tan D (2002). Height and its relationship to refraction and biometry parameters in Singapore Chinese children. Investig Ophthalmol Vis Sci.

[16] Lee DC, Lee SY, Kim YC (2018). An epidemiological study of the risk factors associated with myopia in young adult men in Korea. Sci Rep.

[17] Rahi JS, Cumberland PM, Peckham CS (2011). Myopia over the lifecourse: prevalence and early life influences in the 1958 British birth cohort. Ophthalmology.

[18] Jung S-K, Lee JH, Kakizaki H, Jee D (2012). Prevalence of myopia and its association with body stature and educational level in 19-year-old male conscripts in Seoul, South Korea. Investig Ophthalmol Vis Sci.

[19] Dirani M, Islam A, Baird PN (2008). Body stature and myopia-the genes in myopia (GEM) twin study. Ophthalmic Epidemiol.

[20] Jacobsen N, Jensen H, Goldschmidt E (2007). Prevalence of myopia in Danish conscripts. Acta Ophthalmol Scand.

[21] Javed A, Jumean M, Murad MH, Okorodudu D, Kumar S, Somers VK, Sochor O, Lopez-Jimenez F (2015). Diagnostic performance of body mass index to identify obesity as defined by body adiposity in children and adolescents: a systematic review and meta-analysis. Pediatr Obes.

[22] Oliveros E, Somers VK, Sochor O, Goel K, Lopez-Jimenez F (2014). The concept of normal weight obesity. Prog Cardiovasc Dis.

[23] Park Y, Kim NH, Kwon TY, Kim SG (2018). A novel adiposity index as an integrated predictor of cardiometabolic disease morbidity and mortality. Sci Rep.

[24] Kim JY, Choi J, Vella CA, Criqui MH, Allison MA, Kim NH (2022). Associations between Weight-Adjusted Waist Index and Abdominal Fat and muscle Mass: multi-ethnic study of atherosclerosis. Diabetes Metab J.

[25] Johnson CL, Paulose-Ram R, Ogden CL, Carroll MD, Kruszon-Moran D, Dohrmann SM, Curtin LR. National health and nutrition examination survey: analytic guidelines, 1999–2010. Vital Health Stat 2 2013(161). 25090154

[26] von Elm E, Altman DG, Egger M, Pocock SJ, Gøtzsche PC, Vandenbroucke JP (2007). The strengthening the reporting of Observational studies in Epidemiology (STROBE) statement: guidelines for reporting observational studies. Lancet.

[27] Harb EN, Wildsoet CF (2021). Nutritional factors and myopia: An Analysis of National Health and Nutrition Examination Survey Data. Optom Vis Sci.

[28] Peled A, Nitzan I, Megreli J, Derazne E, Tzur D, Pinhas-Hamiel O, Afek A, Twig G (2022). Myopia and BMI: a nationwide study of 1.3 million adolescents. Obes (Silver Spring).

[29] Qin Z, Du D, Li Y, Chang K, Yang Q, Zhang Z, Liao R, Su B (2022). The association between weight-adjusted-waist index and abdominal aortic calcification in adults aged ≥ 40 years: results from NHANES 2013–2014. Sci Rep.

[30] Li Q, Qie R, Qin P, Zhang D, Guo C, Zhou Q, Tian G, Liu D, Chen X, Liu L (2020). Association of weight-adjusted-waist index with incident hypertension: the rural Chinese cohort study. Nutr Metab Cardiovasc Dis.

[31] Ding C, Shi Y, Li J, Li M, Hu L, Rao J, Liu L, Zhao P, Xie C, Zhan B (2022). Association of weight-adjusted-waist index with all-cause and cardiovascular mortality in China: a prospective cohort study. Nutr Metab Cardiovasc Dis.

[32] Gunes A, Uzun F, Karaca EE, Kalaycı M (2015). Evaluation of Anterior segment parameters in obesity. Korean J Ophthalmol.

[33] Feldkaemper MP, Neacsu I, Schaeffel F (2009). Insulin acts as a powerful stimulator of axial myopia in chicks. Investig Ophthalmol Vis Sci.

[34] Metlapally R, Ki C-S, Li Y-J, Tran-Viet K-N, Abbott D, Malecaze F, Calvas P, Mackey DA, Rosenberg T, Paget S (2010). Genetic association of insulin-like growth factor-1 polymorphisms with high-grade myopia in an international family cohort. Investig Ophthalmol Vis Sci.

[35] Tchernof A, Brochu D, Maltais-Payette I, Mansour MF, Marchand GB, Carreau A-M, Kapeluto J (2018). Androgens and the regulation of Adiposity and Body Fat distribution in humans. Compr Physiol.

[36] Blouin K, Boivin A, Tchernof A (2008). Androgens and body fat distribution. J Steroid Biochem Mol Biol.

[37] Lyu IJ, Oh SY (2023). Association between age at menarche and risk of myopia in the United States: NHANES 1999–2008. PLoS ONE.

[38] Zhang R-H, Yang Q, Dong L, Li Y-F, Zhou W-D, Wu H-T, Li H-Y, Shao L, Zhang C, Wang Y-X et al. Association between vitamin D and myopia in adolescents and young adults: evidence of national cross-sectional study. Eur J Ophthalmol 2023:11206721231161498. 10.1177/11206721231161498 36866629

